# A computational model for biosonar echoes from foliage

**DOI:** 10.1371/journal.pone.0182824

**Published:** 2017-08-17

**Authors:** Chen Ming, Anupam Kumar Gupta, Ruijin Lu, Hongxiao Zhu, Rolf Müller

**Affiliations:** 1 Department of Mechanical Engineering, Virginia Tech, Blacksburg, Virginia, United States of America; 2 Department of Statistics, Virginia Tech, Blacksburg, Virginia, United States of America; 3 Shandong University - Virginia Tech International Laboratory, Shandong University, Jinan, China; Texas A&M University College Station, UNITED STATES

## Abstract

Since many bat species thrive in densely vegetated habitats, echoes from foliage are likely to be of prime importance to the animals’ sensory ecology, be it as clutter that masks prey echoes or as sources of information about the environment. To better understand the characteristics of foliage echoes, a new model for the process that generates these signals has been developed. This model takes leaf size and orientation into account by representing the leaves as circular disks of varying diameter. The two added leaf parameters are of potential importance to the sensory ecology of bats, e.g., with respect to landmark recognition and flight guidance along vegetation contours. The full model is specified by a total of three parameters: leaf density, average leaf size, and average leaf orientation. It assumes that all leaf parameters are independently and identically distributed. Leaf positions were drawn from a uniform probability density function, sizes and orientations each from a Gaussian probability function. The model was found to reproduce the first-order amplitude statistics of measured example echoes and showed time-variant echo properties that depended on foliage parameters. Parameter estimation experiments using lasso regression have demonstrated that a single foliage parameter can be estimated with high accuracy if the other two parameters are known a priori. If only one parameter is known a priori, the other two can still be estimated, but with a reduced accuracy. Lasso regression did not support simultaneous estimation of all three parameters. Nevertheless, these results demonstrate that foliage echoes contain accessible information on foliage type and orientation that could play a role in supporting sensory tasks such as landmark identification and contour following in echolocating bats.

## Introduction

Many bat species perform demanding sensing tasks, such as the detection, localization, and classification of prey and obstacles in dense vegetation based on information provided by highly developed biosonar systems [1, 2]. Compared with bat biosonar, engineered sonar systems used on unmanned aerial vehicles are heavier and bulkier yet they cannot deal with complex targets such as vegetation in forest. For example, navigation based on sonar and other sensors has enabled a blimp to avoid large obstacles, e.g., vertical plates about one meter tall [3], with errors as large as 0.5 m [4]. In contrast to this, bats have been shown to discriminate target range difference between 1 and 3 cm [5].

Vegetation is a prominent feature in the habitat of many echolocating bat species. When capturing prey, echoes from a highly structured background can pose a problem because they can obscure prey echoes and hence reduce hunting success [6]. However, certain vegetation echoes have been shown to provide cues for the identification of flowers with nectar [7] and for the position of fruit in the final localization stage [8].

Similarly, echoes returned from a distributed cloud of scatterers may make it difficult to locate the nearest obstacle or find a passageway through the scatterers. However, foliage echoes could also provide valuable information for navigation by supporting the identification of landmarks [2, 9–11]. It has been shown that Natterer’s bats learn to distinguish conifers from broad-leaved trees [12].

Although vegetation echoes are obviously important to the function of bat biosonar, previous studies have either been limited to a small sample of different foliage types [9, 13–15] or have used a model based on point scatterers [14] that cannot capture the influence of leaf size and orientation on the echoes. In current work, we propose a computational model for foliage echoes that can account for the distribution, size, and orientation of the leaves. Since it has been shown that branches typically contribute little to foliage echoes [16], the ability of the proposed model to capture an extended set of leaf properties should give research into the opportunities and challenges for biosonar that are posed by foliage echoes. The goal of this work is to provide a new powerful tool for creating large ensembles of realistic echoes to mimic different biosonar sensing scenarios that are involved in foliage echoes.

To achieve this goal in a parsimonious fashion, our model uses only three parameters related to the expected values of leaf density, size, and orientation. We demonstrate the utility of this approach by estimating all of these parameters from the model echoes. This allows an assessment of whether all three parameters influence the echo waveforms and could hence potentially impact the operation of bat biosonar systems in foliage—be it as information-bearing or nuisance signals.

## Materials and methods

### Model

**Fig 1:**
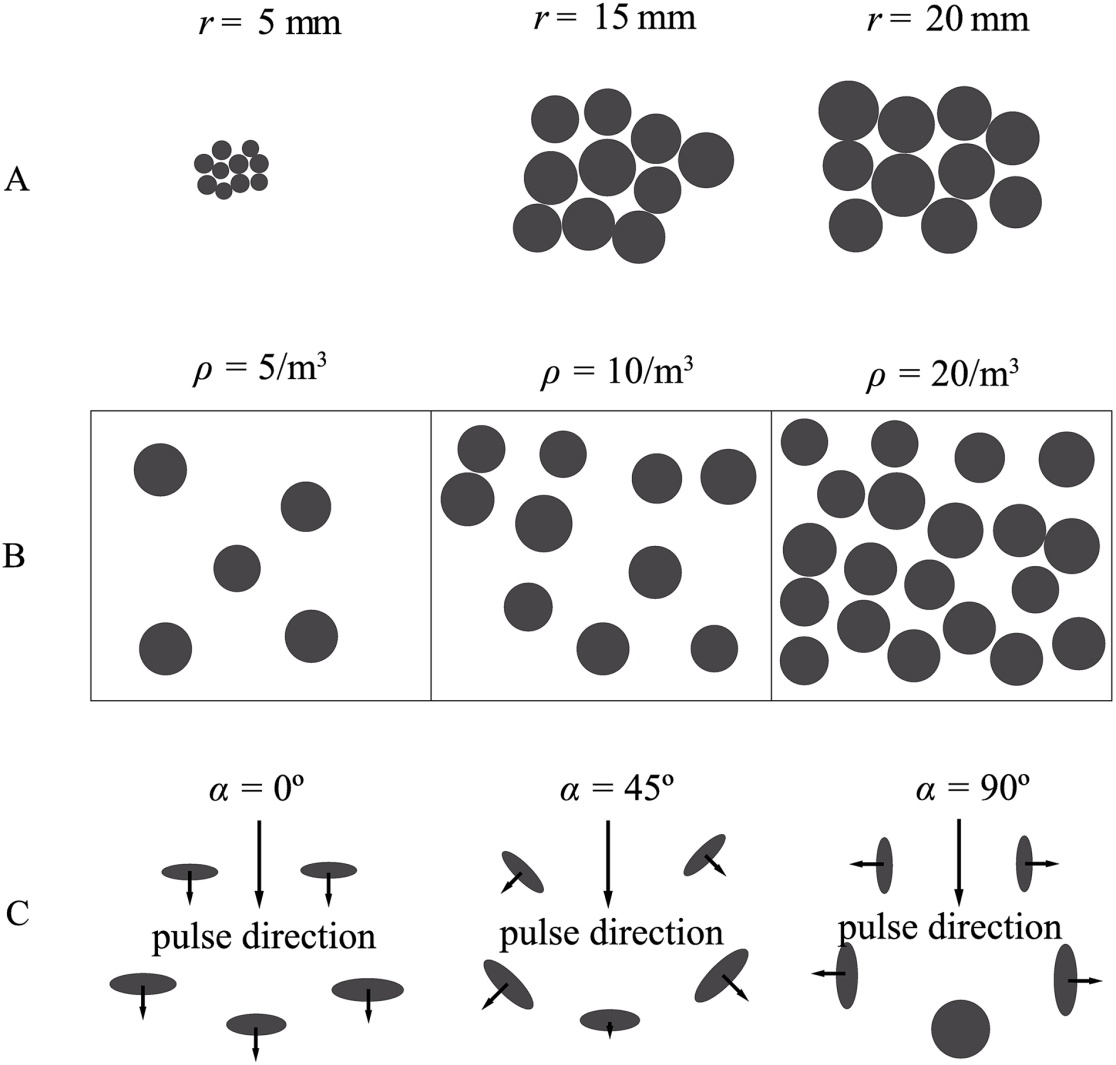
Parameters of the proposed foliage model. A) Expected value of the leaf radius *r*. The radii of all leaves are drawn independently from Gaussian distribution with mean *r* and standard deviation $\frac{r}{10}$. Ten leaves are shown each for mean radii that equal 5, 15, and 20 mm, respectively. B) Leaf density *ρ*. Orthogonal projections on the *xy*-plane of a cubic volume (1 m^3^) filled with 5, 10, and 20 leaves are shown, respectively. The frame represents the borders of the cubic volume. C) Orientations in a leaf sample around mean orientation angles (*α*) of 0°, 45°, and 90°, respectively. The orientation angle is defined as the angle between leaf normal and the direction the sonar is aimed in, orientations are drawn from Gaussian distribution with mean *α* and a fixed standard deviation of 5°.

To arrive at a parsimonious model for the generation of foliage echoes, the following simplifying assumptions have been made in the work reported here:

First, the foliages consisted solely of isolated reflecting facets (“leaves”), i.e., the model did not include any other plant parts such as branches or trunks. Second, multi-path transmission and shading between leaves was ignored. Third, all individual leaf shapes were approximated by acoustically hard, flat, circular discs that are completely characterized by their radius, position, and orientation in space. Fourth, leaves were distributed uniformly in the foliage whereas their distribution in real foliages could be inhomogeneous, e.g., due to branching patterns. Here, all leaves are distributed uniformly inside a rectangular box positioned one meter away from the sonar along the direction of the sonar’s aim. The values of the other two parameters were independently identically distributed Gaussian random variables. To further simplify the model, it was assumed that the standard deviation for the Gaussian distribution of the leaf radii was tied to the mean. It was taken to be one tenth of the mean. In this way, leaves that were larger on average also varied more in size. The standard deviation of the orientation angle of the leaf was fixed at a value of 5°. Under these assumptions, the simplified model foliages can be described by three parameters: the mean leaf radius *r*, leaf density *ρ* (number of leaves per cubic meter) and mean leaf orientation *α* (Fig 1). The angle to describe leaf orientation was chosen as the angle between the surface normal of the leaf and the pointing direction of the sonar.

For plane waves incident on an acoustically hard circular disc at an angle *ζ* with the surface normal, the scattered field, *V*^*s*^, is given in the far field as

$$\begin{array}{c} V^{s}∼\frac{e^{ikd}}{kd}S, \end{array}$$ (1)

where *d* is the distance between sonar and disc, *k* is the wave number, and *S* is the far field coefficient. The far field coefficient *S* can be expressed as an infinite sum of spheroidal wave functions as follows [17]:

$$\begin{array}{c} S=2i\sum_{m=0}^{\infty }\sum_{n=m}^{\infty }\frac{\epsilon_{m}}{{\tilde{N}}_{mn}}\frac{R_{mn}^{{\left(1\right)}^{\prime }}\left(-ikr,i0\right)}{R_{mn}^{{\left(3\right)}^{\prime }}\left(-ikr,i0\right)}S_{mn}\left(-ikr,\cos\zeta \right)S_{mn}\left(-ikr,\eta \right)\cos m\phi , \end{array}$$ (2)

where *r* is the radius of the disc, $k=\frac{2\pi }{\lambda }$ the wave number, *S*_*mn*_(−*ikr*,*η*) are oblate spheroidal angle functions of the first kind, of order *m*, and degree *n*, $R_{mn}^{(1{)}^{\prime }}(-ikr,i0)$ is the derivative of the oblate radial functions of the first kind, of order *m*, and degree *n*, $R_{mn}^{(3{)}^{\prime }}(-ikr,i0)$ is the derivative of the oblate radial functions of the third kind, order *m*, and degree *n*, *ϵ*_*m*_ is the Neumann symbol, (*η*,*ξ*,*φ*) is the position of observation point in oblate spheroidal coordinates, and ${\tilde{N}}_{mn}$ is a normalization constant.

**Fig 2:**
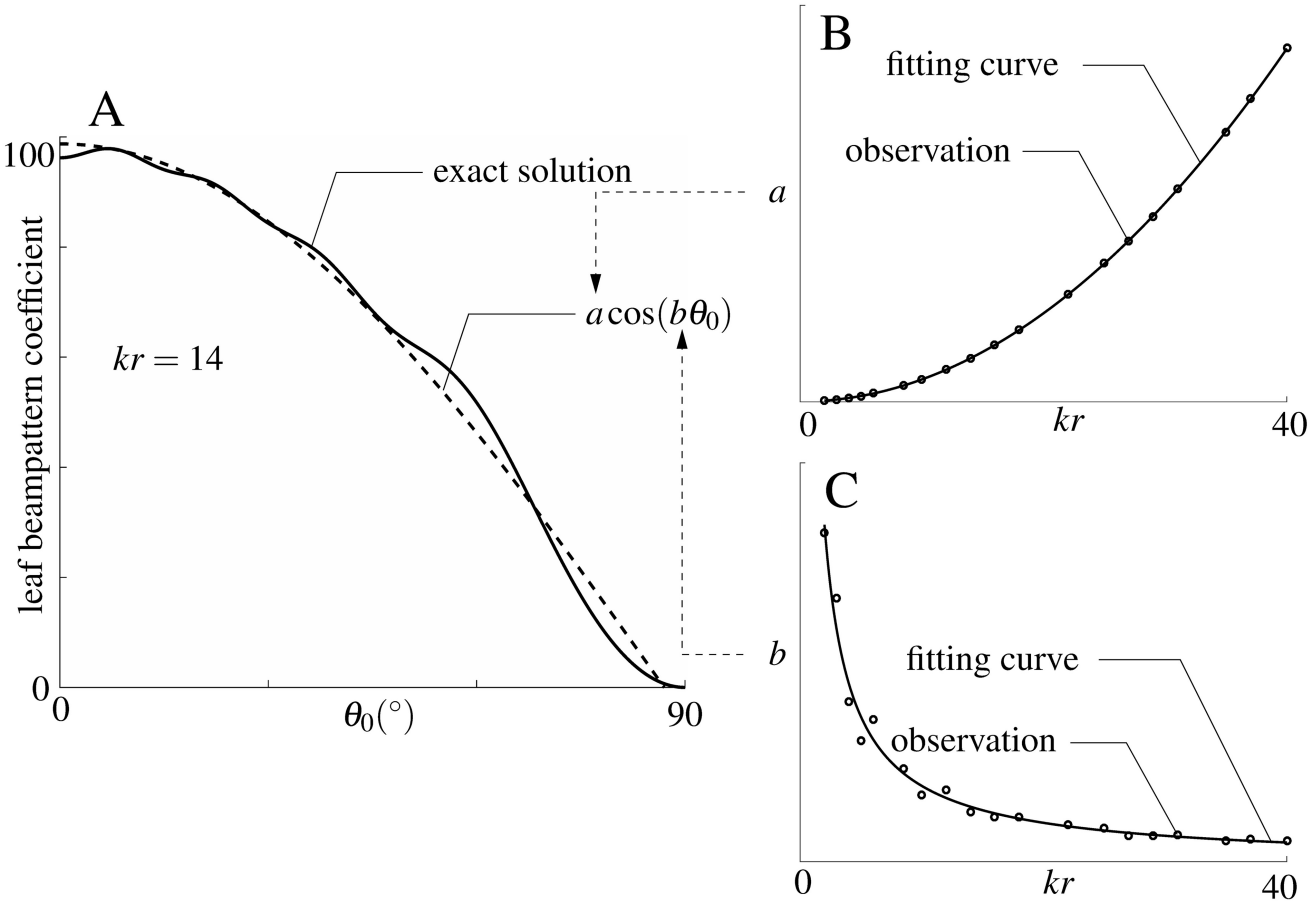
Approximation to the scattering beampattern of a disc. A) evaluation of the truncated infinite series for the leaf beampattern coefficient (Eq 2) and its approximation using a cosine function; solid line: series evaluation; dashed line: cosine fit (*a*cos(*bθ*_0_)). The result from the infinite series and the cosine approximation are shown as a function of incident angle *θ*_0_ (0° to 90°) for the case of *kr* = 14, where *k* is wave number and *r* is the leaf radius. B) and C) Fitting curves used to determine the amplitude *a* and angular frequency *b* of the cosine fit as a function of *kr*. Open circles: parameter values determined from evaluating a truncated version of the infinite series; solid lines: curve fitted to the data points marked with the open circles.

To evaluate the scattered field of discs with different radii over the frequency range of interest here (60 to 80 kHz, modeled after the second/strongest harmonic in the biosonar pulses of the greater horseshoe bat, *Rhinolophus ferrumequinum* [18]), the following procedure was used: For each value of *kr*, the numerical values of the far-field coefficient *S* (Eq 2) were calculated for 1000 evenly spaced values of incident angle between 0° to 90° (Fig 2A) by numerical evaluation of a truncated version of the series in Eq 2 [19]. The series coefficients decay exponentially and were truncated based on a magnitude threshold [19]. Since calculating the scattered field of a single disc in this way takes several days on a standard work station, a more time-efficient approximation of the numerical solution with cosine functions was used to obtain all the scattered field values for the different disc diameters and incident angles. The cosine function used for this approximation was of the form *a*cos(*bθ*), where the amplitude *a* and the angular frequency *b* were used as fitting parameters. A nonlinear least squares method based on a trust-region-reflective algorithm [20] was used to accomplish this fit. These fits resulted in data sets containing values for the parameters *a* and *b* for each value of the product *kr* (Fig 2B and 2C). In order to be able to arrive at values for the parameter *a* and *b* for any value of *kr* without much computational cost, power functions were fitted to the relationships between the parameters and *kr*. This fit was carried out using the same method as described above. The fitting functions used were $a(kr)=\frac{1}{2}(kr{)}^{2}+0.7$ (i.e., a second order polynomial), and *b*(*kr*) = 0.4(*kr*)^−0.9^ + 1, respectively (Fig 2B and 2C).

**Fig 3:**
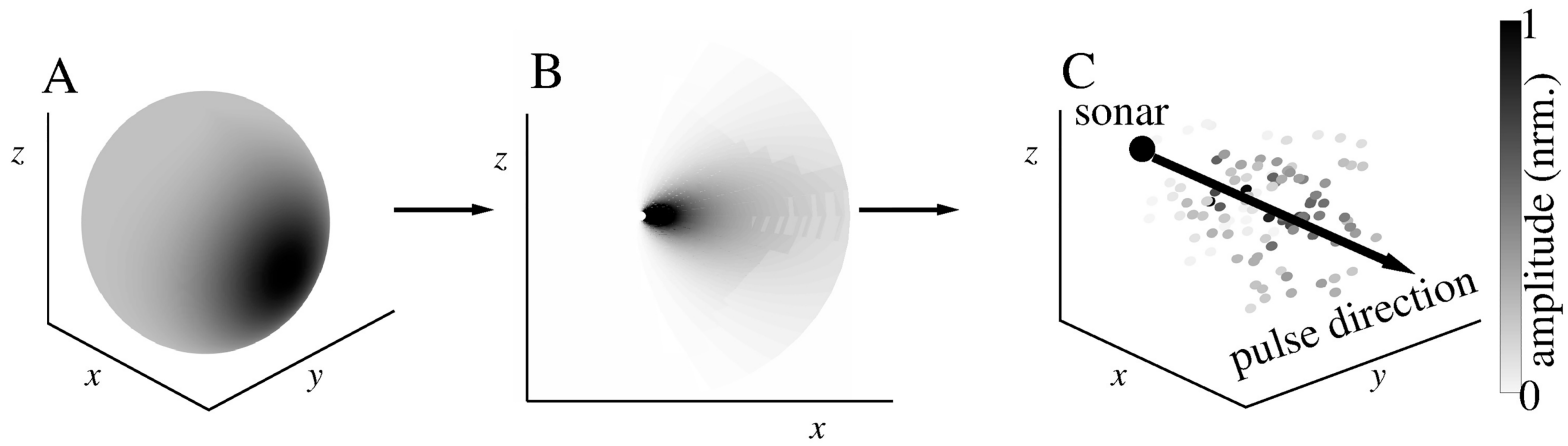
Biosonar beampattern model. A) distribution of beam gain amplitude over direction (-3 dB beamwidth 30°); B) distribution of sonar gains in the *xz*-plane showing directionality gains and spreading losses; C) total (emission and reception) sonar gain mapped on a cluster of leaves representing leaves in a foliage. The axis of maximum beamgain of the sonar is aligned with the +*x* direction. Leaves are uniformly distributed. Beamgain amplitudes are normalized and encoded by gray scale.

The shape of the bat biosonar beampattern was approximated by the product of two Gaussians, one a function of azimuth and the another a function of elevation. The sonar was assumed to be monostatic, i.e., emitter and receiver were in the exactly same position which can be justified since the small sizes of bat heads (a few centimeters diameter at most) will not result in a substantial parallax when looking at targets at a distance of one meter or more. The standard deviations of the Gaussian functions used to model the beampattern in azimuth and in elevation were fixed at the same value of 17.2° corresponding to a -3 dB beamwidth of approximately 30°. The beampatterns’ direction of maximum gain was aligned with the normal to one of the surfaces of the rectangular domain in which the leaves were distributed (Fig 3).

**Fig 4:**
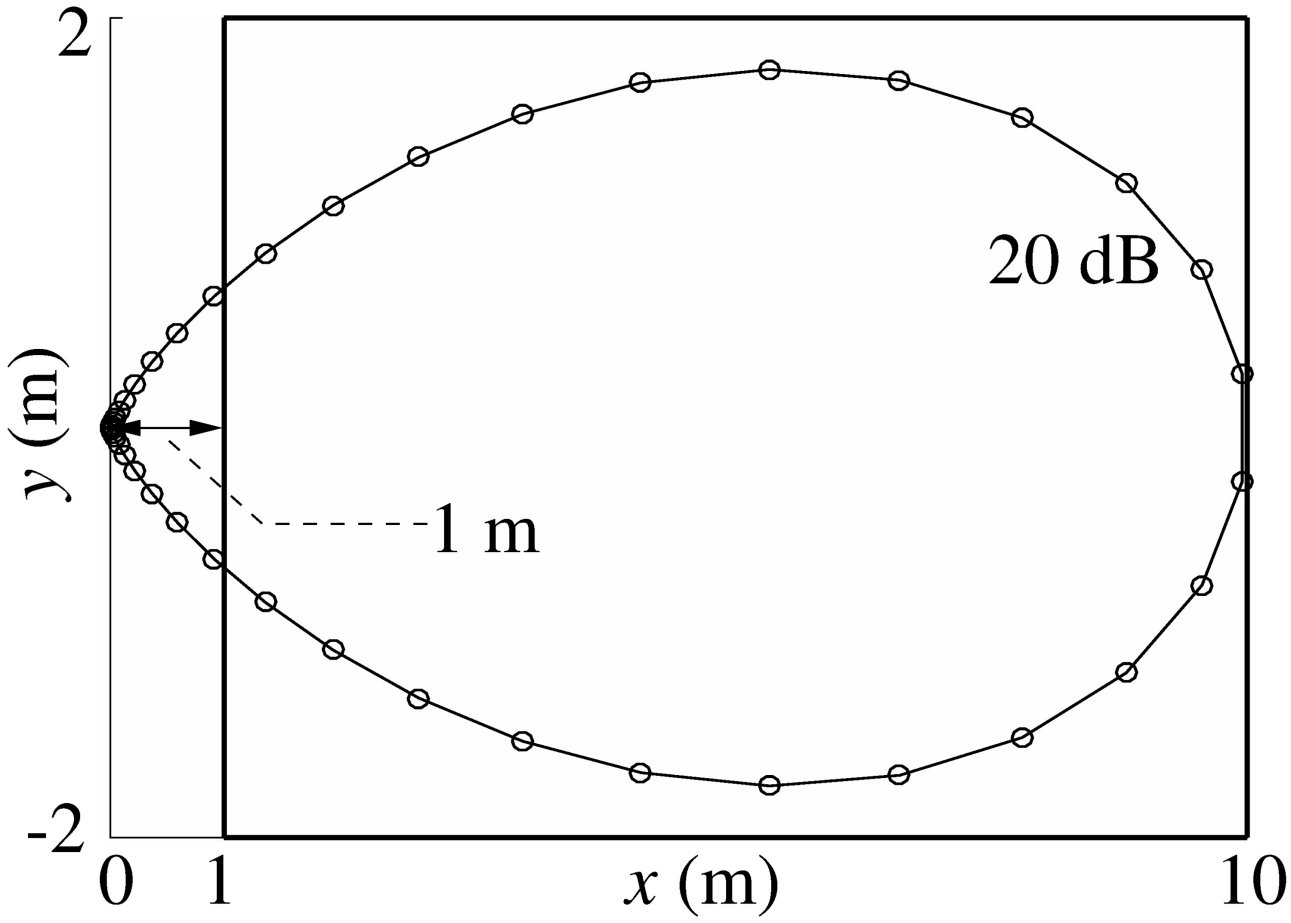
Determination of the foliage domain covered by the computations. Iso-gain contour of a sonar beam cross-section for a sound pressure level of 20 dB SPL assuming a source level of 100 dB SPL at 10 cm distance. Emission and reception losses were both considered in these calculations. The Boundaries of the foliage domain cuboid in the *xy*-plane (solid-line rectangle) were positioned to contain the entire 20 dB SPL iso-gain surface and were rounded up to the next integer multiple of 2 m. The distance between sonar and the cuboid was set to 1 m to ensure the far-field assumption made in the calculation of the scattering from leaves was valid.

The boundaries of the cuboid-shaped spatial domain over which discs (leaves) were randomly placed to be included in the calculation of the echoes were determined based on the expected maximum gain for the respective position. Positions for which losses due to beamgain and spreading amounted to a drop of more than -80 dB in amplitude were not included (Fig 4). This reasoning was based on the dynamic range of the sonar in greater horseshoe bats for which an emission level of around 100 dB SPL in a distance of 10 cm [21] and a hearing threshold around 10 dB SPL [22] have been reported and the assumption that the target strength of the leaves typically does not exceed -10 dB substantially (the target strength of a disk with 4 cm diameter at a frequency of 75 kHz).

The distance between sonar and leaf domain was set to 1 m in order to ensure that the far-field assumption in the calculation of leaf beampattern is valid. The far-field distance for emissions from greater horseshoe bats with call frequency 83 kHz and noseleaf width 8.1 mm [23] would be 3.2 cm. For the ears, an ear length of about 2.2 cm would results in a transition to the far-field at approximately 24 cm [24].

The sonar pulse was assumed to have a power spectrum that was flat between 60-80 kHz. To acquire the output signal which was an impulse response of ensonified leaves in the model, the frequency domain signal of each leaf in 60-80 kHz range was first calculated with the sonar beampattern and the approximated leaf beampattern according to the leaf’s position and orientation relative to the sonar. Responses at other frequencies were set to zero. Then the frequency responses of all leaves were superpositioned. Finally, the inverse Fourier transform was applied to obtain the time domain signal.

### Estimation of foliage parameters

Lasso regression was used to estimate the values of the foliage parameters from the resulting echoes. Hence, the parameter estimates were linear combinations of the weighted echo features. Finding the weights for the best-trained model was accomplished by minimizing the sum of the parameter estimation errors in a least-square sense over the observations subject to a constraint on the sum of the weight values. The sum of the absolute values was limited to a maximum value. This maximum value, called a “tuning parameter” can be used to control number of features used. The best value for the tuning parameter was determined by virtue of a cross validation approach [25].

**Fig 5:**
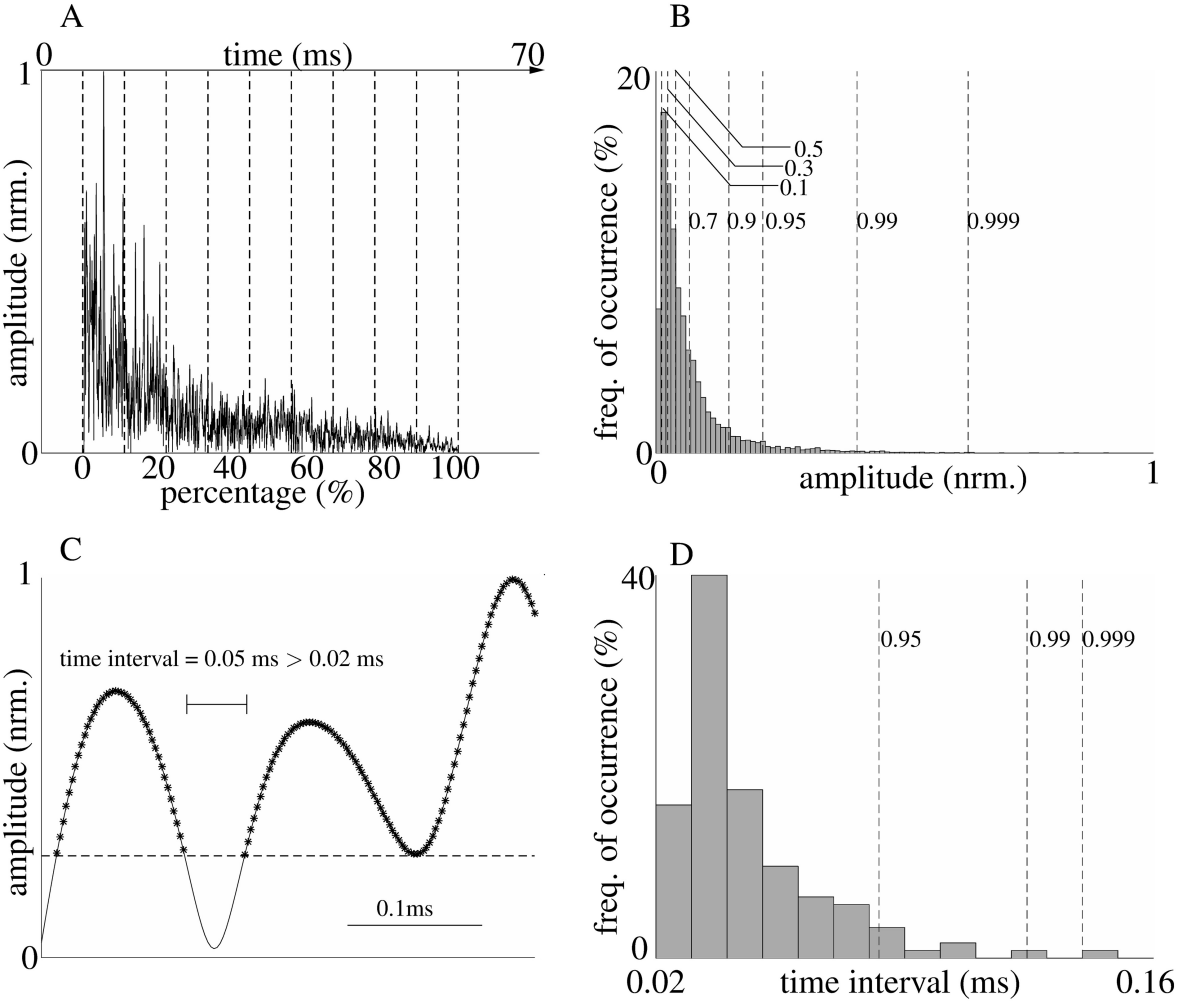
Part of the statistical echo features. A) Echo envelope evenly divided into 10 time intervals. B) [0.1, 0.3, 0.5, 0.7, 0.9, 0.95, 0.99, 0.999] quantiles of the amplitude above a threshold (one tenth of the maximum value in the last time window, i.e., 90% to 100%). C) Example of a time interval longer than 0.02 ms in zoomed-in view of the envelope. The time intervals are time difference of two adjacent points with values larger than the threshold mentioned above. Find all time intervals and keep the ones longer than 0.02 ms. D) [0.95, 0.99, 0.999] quantiles of the time intervals longer than 0.02 ms. All figure are derived from the same single example echo.

A total of 40 features were extracted from each echo for the purpose of estimating the underlying parameters of the foliage model (Fig 5). One group of these parameters contained measures of the distribution of the envelope magnitude values taken over the entire echo, since both of the three parameters would contribute to envelope magnitudes. Similarly, measures of how the peaks in the echo amplitude were distributed in time over the entire echo were used as a basis for estimating the foliage model parameters, which are more related to leaf density and may help estimation by isolating the impact of density from that of the other two parameters on echoes. The remaining features were measures derived from the distribution of envelope magnitudes within ten time windows of even length that spanned the entire duration of the echo.

For features derived from the echo envelope amplitudes, a threshold of one tenth of the maximum envelope magnitude in the last time window was set to exclude features associated with very small magnitude values from these calculations. The following features were derived from the magnitudes of the envelope of the entire signal: area under the envelope, quantiles (0.1, 0.3, 0.5, 0.7, 0.9, 0.95, 0.99, 0.999) of magnitude values that were larger than the magnitude threshold, central moments (2nd to 5th) of the above-threshold magnitude values, the same four central moments for time intervals longer than a 20 *μ*s threshold. The time-differences were measured between neighboring points in the signal envelope that were higher than the threshold, and a set of quantiles (0.95, 0.99, 0.999) of those selected time intervals. The quantiles were used to describe the shape of the probability density function (pdf) of those magnitudes. The 2nd central moment is the variance and the scaled version of third central moment is skewness, a measure of the lopsidedness of the distribution. The scaled version of fourth central moment is kurtosis and serves as a measure of the heaviness of the tail of the distribution. Features calculated within the 10 time windows were: the number of magnitude values larger than the threshold in each window and mean value of the magnitudes above the threshold in each window.

Cross validation was used to find the tuning parameter and avoid overfitting. It was carried out using 80% of the echoes. This cross-validation echo data set was divided into 10 subsets of equal size. One subset was excluded and the lasso model was fitted to the remaining nine subsets of the echoes. Then the model was tested on the excluded subset. The process was repeated 10 times in total where each time a different subset was excluded. The estimation errors made over all these 10 tests were summed and used to determine the value of tuning parameter that resulted in the minimum error.

With this cross validation followed by the lasso regression itself, foliage parameter estimates were carried out for the following three scenarios: (i) estimation of a single unknown foliage parameter with the other two parameters fixed, (ii) estimation of one foliage parameter with the second parameter fixed and the third parameter left to assume unknown and variable values, and (iii) estimation of one parameter with the other two parameters remaining unknown and subject to change.

In all these estimation scenarios, the following values were used for the known/fixed parameters: leaf density 100/m^3^, mean orientation angle 7°, and mean leaf radius 15 mm. Parameters left unknown were drawn randomly from the values in the following sets in each echo: leaf density [20, 100, 200, 300, 500]/m^3^, mean orientation angle [0, 20, 40, 60, 80]°, and mean leaf radius [7, 10, 13, 17] mm. Parameters to be estimated were left to take any integer within the following intervals: leaf density [20, 500]m^3^, mean orientation angle [0, 90]°, and mean leaf radius [5, 20]mm. For the estimation of a single unknown parameter, 100 echoes each were generated for 100 different parameter combinations. As described above, 80 of these echoes were used for training and the remaining ones were used to test the lasso model. For the estimation of parameters in other situations, 500 echoes were generated from 100 parameter combinations with 5 echoes from each. Among those echoes 400 were used in training, and the remaining 100 echoes were used in testing.

### Field echo recordings

The impulse responses from real trees were recorded for comparison with the simulated echoes. Recording of the echoes in the field was carried out using a biomimetic sonar head. The sonar head used a single electrostatic ultrasonic loudspeaker (Series 600 open face ultrasonic sensor, SensComp, Inc., Livonia, MI, USA) with a two-sided -3 dB beamwidth of 10° at 50 kHz. A power amplifier (AA-301HS, A.A. Lab Systems Ltd. Ramat-Gan, Israel) was used to drive the loudspeaker. Two MEMS capacitive microphones (SPU0410LR5H, Knowles Electronics, LLC. Itasca, IL USA) mounted on pre-amplifier boards (Momimic, Dodotronic, Rome, Italy) were used for signal reception. An A/D and D/A conversion board (NI-6351, National Instruments Corp., Austin, TX USA) was used in the setup and provided 16-bit resolution and 500 kHz sampling rate for digital-to-analog and analog-to-digital conversion. The sonar head was mounted on a tripod, the height of which above ground was adjusted according to different trees to let the transmitted signal hit the foliage at an approximately normal incident angle. The emitted signal consisted of a 5-ms-long linearly modulated chirp covering a frequency band from 20 to 100 kHz. The experiments were done on Virginia Tech’s campus, and no endangered or protected species were involved. No specific permissions were required.

### Comparison of simulation results with field recordings

The echoes of two different tree species were recorded for comparison to the simulated echoes: Japanese maple (*Acer palmatum*) and coniferous tree, arborvitae (*Thuja occidentalis*). Echoes were obtained from one tree per species. 400 echoes (200 per microphone) were collected per viewing angle with a total of 5 viewing angles for each tree. 30 leaf samples [26] were collected from 4 branches at different heights for Japanese maple. The mean leaf area over 30 leaves was obtained by calculating the area of each leaf after scanning it and counting the dark (green) pixels. The equivalent leaf radius that produced the same area for a circular disk was used to determine the mean leaf radius of the model, which was 1.46 cm. Mean orientation angle and density were decided based on observation. The foliage of Japanese maple consisted of dense leaves with mean orientation angle about 45°, and thus the density was set to 5000/m^3^. The arborvitae had foliage with forms of flat sprays with scale-like leaves; in the model, 1 mm, 45°, and 10000/m^3^ were used to represent its mean leaf radius, orientation angle, and leaf density, respectively. The measured echoes were first filtered with passband 20–110 kHz, then cross-correlated with emitted signal to acquire the impulse response, and filtered again with passband 60–80 kHz to match the frequency range in simulation.

In the simulation that were designed to mimic the measured tree foliages, the length of the foliage domain along the sonar’s line of sight was set to match the pulse duration observed in the field recordings. The distance between the sonar head and nearest leaf was determined from the first point in time where the echo amplitudes clearly exceeded the noise amplitudes. The same distance was then used in the respective simulations. The -3 dB sonar beamwidth for these simulations was set to 10° to match the beamwidth of the experimental setup.

For a quantitative comparison between the simulated and the measured foliage echoes, each echo was divided into three time windows of equal length. In each of these windows, a histogram estimate for the probability density function (pdf) of the echo amplitudes was obtained. The difference between pdf estimates for simulated and measured echoes was quantified using the Bhattacharyya distance [27]. The values of the Bhattacharyya distance range from zero to infinity. The smaller the distance is, the better two pdfs match each other. In order to provide a reference for judging these difference, the differences obtained for simulated versus measured pdfs were compared to the differences found within the simulated and measured data sets.

## Results

### Comparison of real echoes and simulation

**Fig 6:**
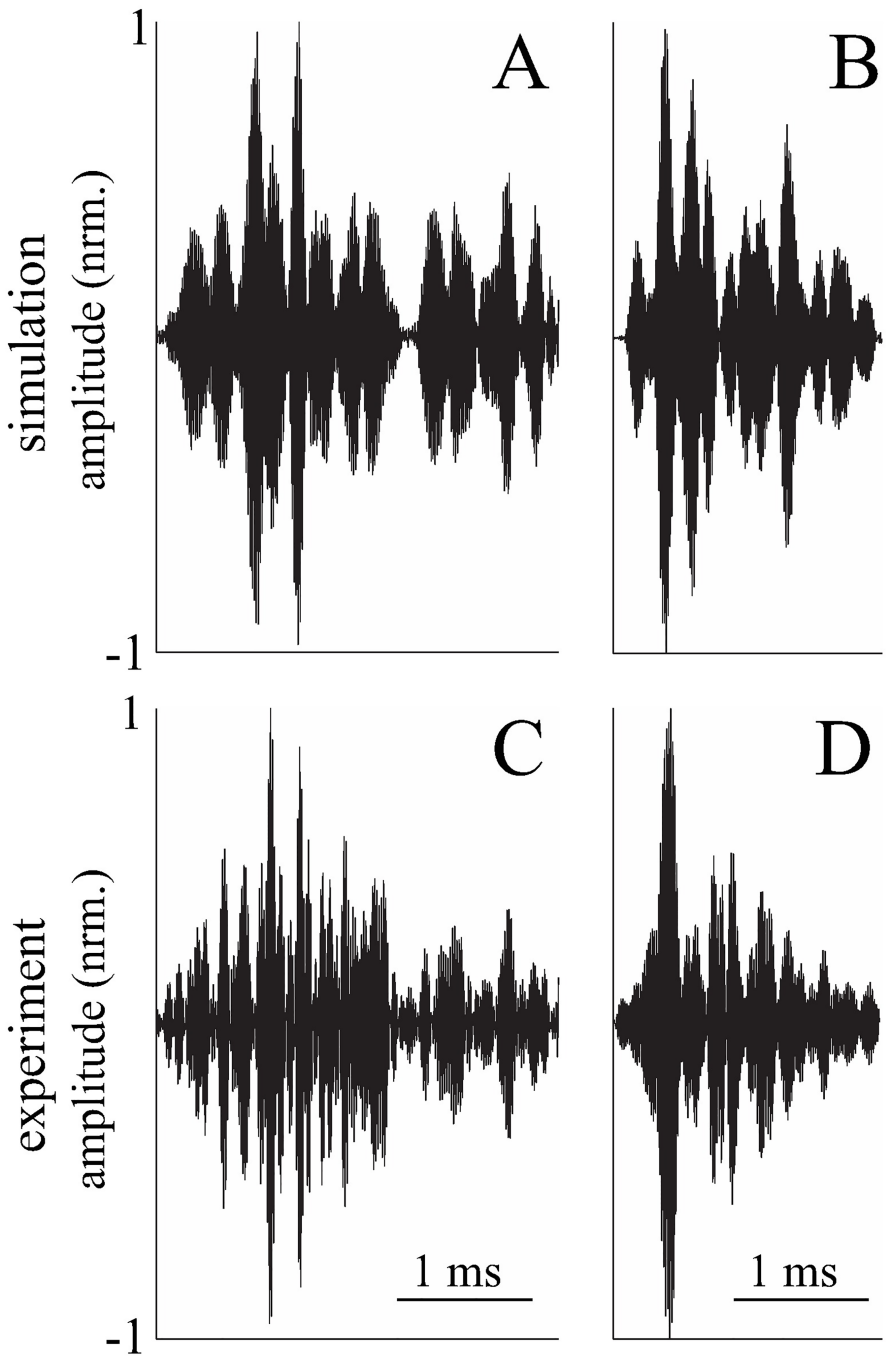
Comparison between examples of simulated and measured echoes. A-B) Simulated echoes: A) with parameters similar to the Japanese maple specimen: mean leaf radius 1.46 cm, density 5000/m^3^, and mean orientation angle 45°. B) with parameters similar to the arborvitae specimen: mean leaf radius 0.1 cm, density 10000/m^3^, and mean orientation angle 45°. C-D) echoes measured in the field: C) Japanese maple, D) arborvitae. All echo amplitudes were normalized to their respective maximum.

**Fig 7:**
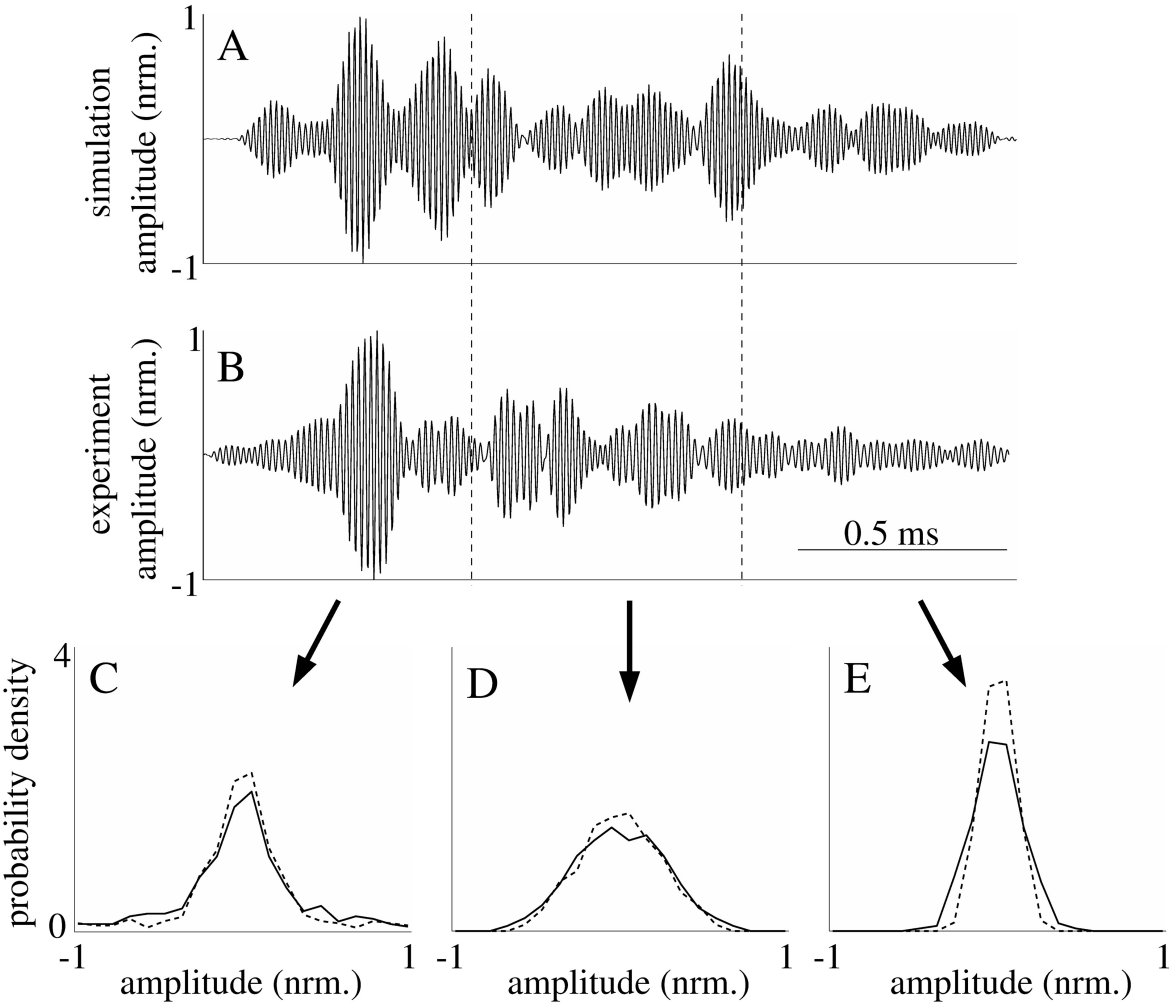
Comparison of probability density functions for the amplitudes of simulated and measured echoes. A) simulated echo produced by a model with parameters adjusted to mimic arborvitae. B) echo from the same species measured in the field. The simulated and measured echoes were segmented into three time windows of equal length, the probability density functions for the amplitudes in each segment are shown in C), D), and E). Solid lines: simulated echoes; dashed lines: measured echoes.

The waveforms of the measured and simulated echoes were found to be qualitatively very similar to each other for the two tree species/specimens studied (Fig 6). This impression was confirmed by quantitative comparisons of the first-order probability density functions obtained for the signal amplitudes. The Bhattacharyya distances used for this purpose were fairly similar for comparisons within the simulated and measured echoes on the one hand and comparisons between these two data groups on the other. For example, in the central time windows of the echoes from the arborvitae, the average Bhattacharyya distance between pdfs of simulated echoes was 0.03 (±0.02 standard deviation, *N* = 5) that between pdfs of measured echoes was 0.04 (±0.02 standard deviation, *N* = 5), and that between simulated and measured echoes was 0.03 (±0.02 standard deviation, *N* = 5, Fig 7).

### Time-varying echo properties

**Fig 8:**
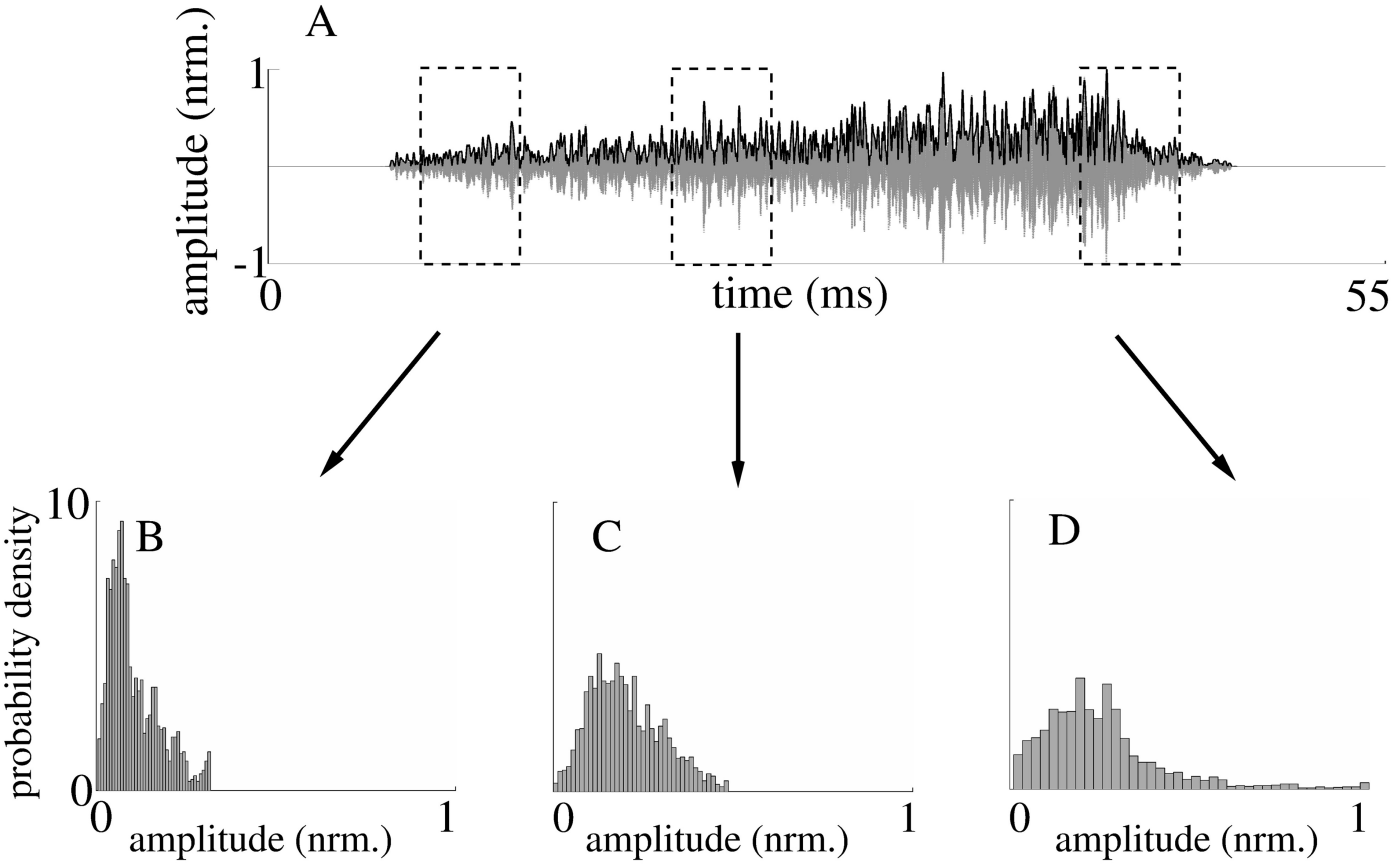
Example of time-varying echo and its probability density functions in three different windows. A) simulated echo waveform with envelope (black) computed without inclusion of spreading losses. B), C), and D) probability density function for the three time windows shown in A).

**Fig 9:**
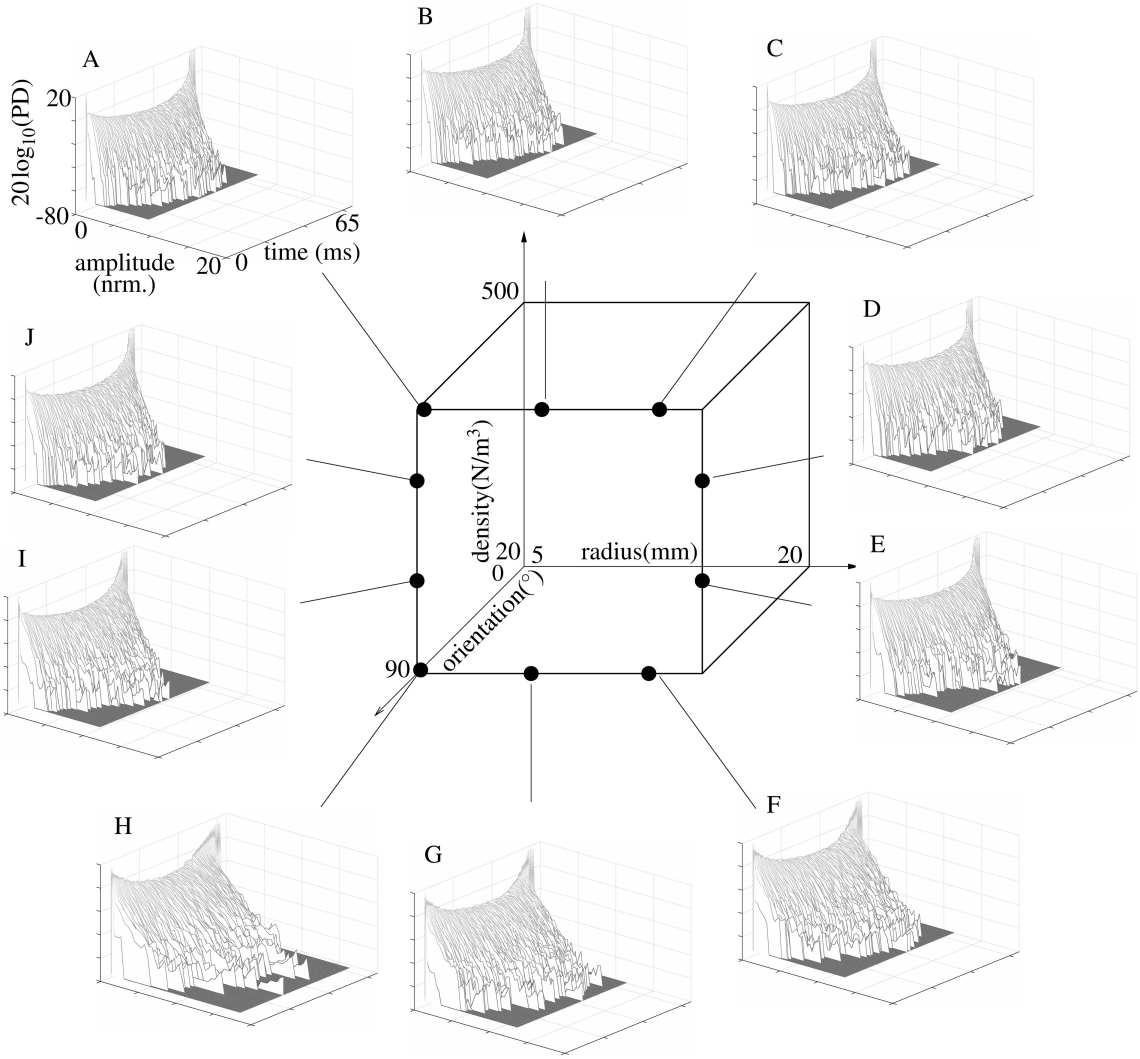
Time-varying nature of the foliage echoes. Logarithm of the probability density (20 log_10_(PD)) of the echo envelope amplitude as a function of time. The data set used for each plot contains 100 echoes each of which was normalized to the the maximum root-mean-square level within the respective data set. A-J) probability density functions for different points in the foliage models feature space (center).

The most visible time-variant property of the echoes (simulated or measured) was a decay in echo amplitude with increasing time due to geometric attenuation. That was reflected by amplitude probability density functions with decreasing spread over time (Fig 7). However, after the effects of the geometric spreading losses were removed from the echoes, the resulting amplitudes remained time-variant but with a reverse dependence where the spread of the amplitude probability functions tended to increase with time (Figs 8 and 9). This remaining non-stationary behavior of the echoes was quantified using the Bhattacharyya distance as a difference measure for probability density functions associated with different times during the echoes. This analysis provided evidence that the time-variant behavior of the probability density functions was linked to the properties of the model foliages: For example, among all the Bhattacharyya distances obtained between probability density functions obtained for different time windows positioned within the central 80% of the echo durations, the 95%-percentile of the differences increased by 40% (from 0.1 to 0.14) as the leaf density was increased from 20/m^3^ to 500/m^3^. Hence, in this case, the 5% largest differences between the probability density functions increased as the model foliages (mean leaf radius: 5mm, mean leaf orientation: 90°) became denser. Likewise, the Bhattacharyya distances were also found to depend on leaf orientation: As the average leaf orientation was changed from 0° to 90°, the 95%-percentile of the Bhattacharyya distance was reduced by 50% from 0.24 to 0.12. Hence, the time variance was greater when the leaves were oriented with respect to the sonar’s light of sight.

### Parameter estimation

**Fig 10:**
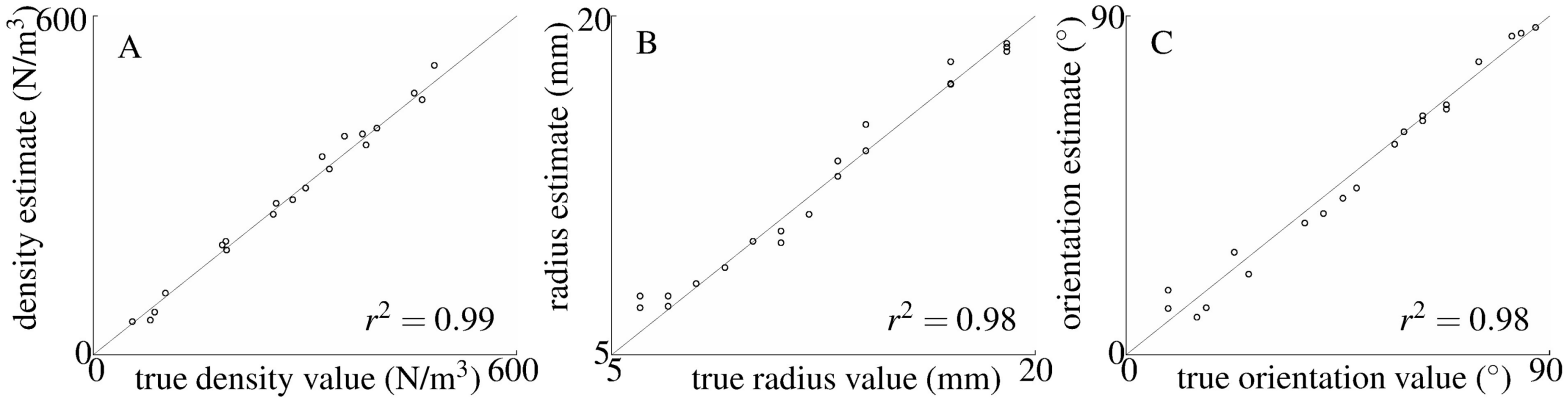
Estimation of a single model parameter with the other two parameters fixed and known. Estimates of A) leaf density *ρ*, B) mean leaf radius *r*, and C) mean leaf orientation *α*. Whenever a parameter was assumed to be fixed at a known value, leaf density, mean leaf radius, and mean leaf orientation were set to 100/*m*^3^, 1.5 cm, and 7°, respectively. The coefficient of determination is indicated in the bottom right corner for each estimation.

**Fig 11:**
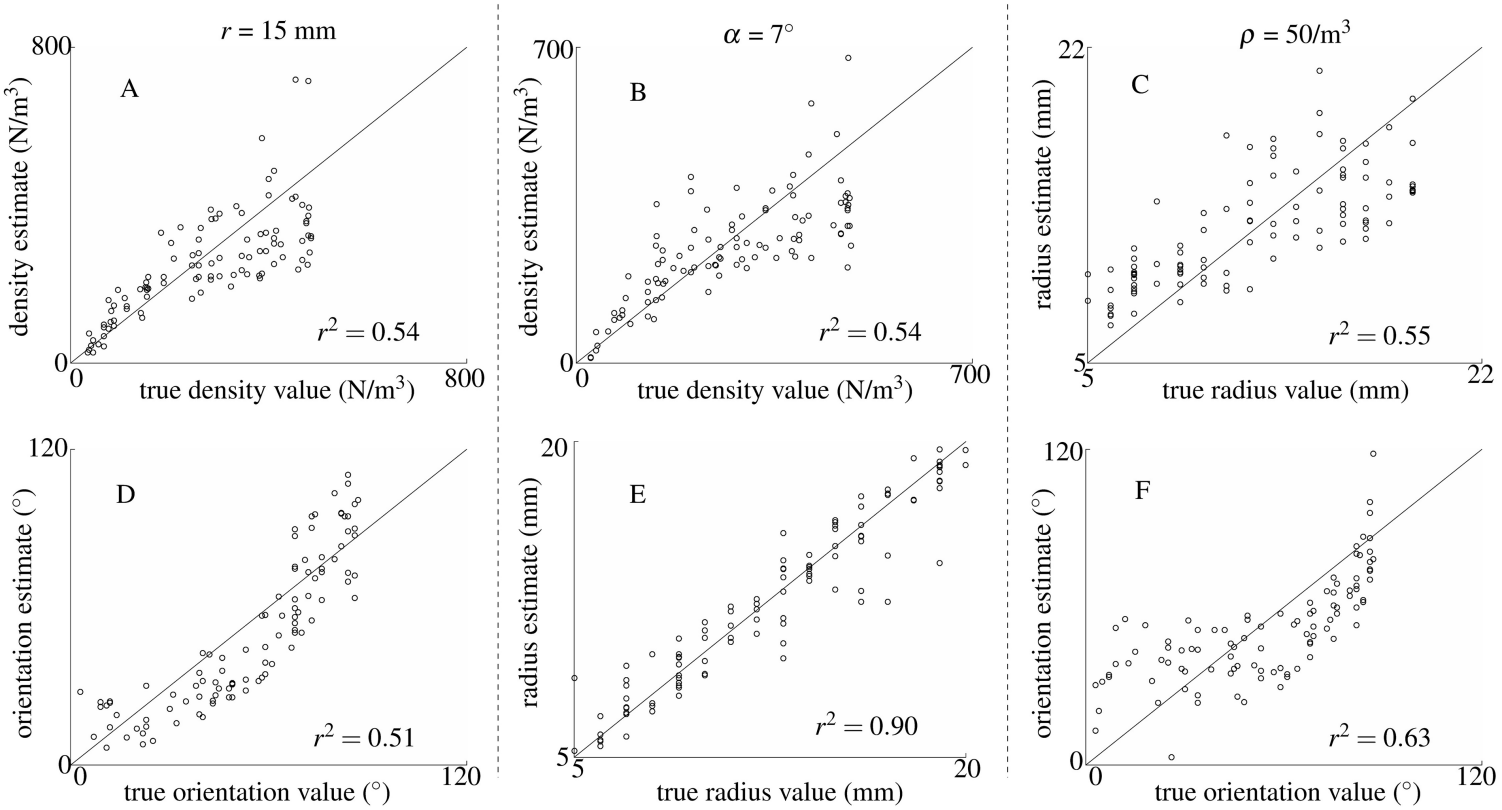
Estimates of a single model parameters with one known and one unknown parameter. A) estimates of leaf density *ρ* with mean leaf radius *r* fixed, B) estimates of leaf density *ρ* with average leaf orientation *α* fixed, C) estimates of leaf radius *r* with leaf density fixed, D) estimates of average leaf orientation *α* with leaf radius *r* fixed, E) estimates of leaf radius *r* with average leaf orientation *α* fixed, F) estimates of average leaf orientation *α* with leaf density *ρ* fixed. Whenever a parameter was fixed to a known value, 100/*m*^3^, 1.5 cm, and 7° were used for leaf density, mean leaf radius, and mean leaf orientation, respectively. Whenever a parameter was left unknown and free to change, it was selected randomly from the values in the following sets for each echo: leaf density [20, 100, 200, 300, 500]/*m*^3^, mean orientation angle [0, 20, 40, 60, 80]°, and mean leaf radius [7, 10, 13, 17] mm. The coefficient of determination is indicated in the bottom right corner for each estimation.

It was found that accurate estimation of a single unknown foliage parameter was readily achievable with the lasso regression method employed (Fig 10). For all three parameters of the model, the estimates were highly correlated to the actual values (*r*^2^ values: 0.99, 0.98, and 0.98 for leaf density, mean leaf orientation, and mean leaf radius, respectively). For the estimation of one foliage parameter where one of the other two parameters is known and the other remains unknown, all six possible scenarios yielded correlations between the true parameters and the estimates that were lower than those obtained for single unknown parameters with most *r*^2^ values falling between 0.5 and 0.6 (Fig 11). An exception was the estimation of leaf radius with known average orientation and unknown leaf density, where an *r*^2^ value of 0.9 was reached (Fig 11E). Finally, estimation of one parameter was attempted with the other two parameters unknown, these estimates were poorly correlated with the actual parameters and hence did not provide much useful information of foliage features.

## Discussion

The novelty of the foliage model presented here lies in the use of discs to approximate the acoustic scattering behavior of the leaves. This introduces two additional parameters, leaf size and leaf orientation, that are not needed in models based on omnidirectional point scatterers [14]. While addition of these parameters makes the model more complicated, both of these foliage parameters could be of importance to the sensory ecology of bats: Being able to estimate the average size of the leaves in a foliage could help bats to identify the type (e.g., the species) of trees or bushes they encounter. The ability to tell the type of a foliage from the echoes could, in turn, support the recognition of individual trees or bushes that may serve as landmarks for navigation. Similarly, the ability to identify foliage types could help the bats to find their food if certain foliage types are more likely associated with the presence of food [7, 8] than others. Leaf orientation could play an important role in guiding the bats’ flight path in close proximity to foliage [28], e.g., when the animals are following the contour of a vegetation edge. Since the leaf normals in a foliage are likely to be oriented towards the surface of the foliage, being able to tell the average direction of the leaf surface normals could be a convenient way to determine the orientation of a foliage contour and control the direction of a flight path that follows the contour without collision.

The results presented here demonstrate that estimation of foliage parameters (leaf density, average leaf size and orientation) from the echo waveforms is possible using echo features that could also be accessible to bats in a similar form. However, with the method used here (lasso regression), highly accurate estimation was only possible for a single unknown parameter. Estimation of two unknown parameters yielded results that could still be useful, but provided a much lower accuracy than was the case for a single parameter. Since there is no reason to believe that present results constitute an upper bound on the achievable performance, it remains possible that bats may be able to perform vastly better than the current pilot results. But even if this is not the case, the level of estimator performance demonstrated here could be very helpful to bats in densely vegetated habitats. For example, a bat may have sufficient *a priori* knowledge about the leaf density and average leaf size in the foliage of its habitat. A bat armed with such accurate *a priori* information would be able to get precise estimates of average leaf orientation that it could use to follow foliage contours within a known habitat. For landmark identification in an uncertain location, bats may be able to determine the orientation of the foliage surface and hence the average orientation of the leaf normals through other means, e.g., by looking at the foliage surface from different directions. Once the average orientation of the leaves is known, the animals could use this *a priori* information to obtain estimates for leaf density and average leaf size to identify a known landmark tree or bush by its foliage type.

Spreading losses for ultrasonic waves traveling in a three-dimensional medium impose strong time-variant signatures onto echoes that originate from a foliage where the reflectors (leaves) are spaced over a wide range of distances from the sonar. However, these effects do not reveal much about the target other than the range at which a certain component of the target’s impulse response has originated. However, the same information is already available from the time of flight in a much more reliable fashion since echo amplitude depends on transmission losses as well as target strength whereas time of flight depends only on target range. Hence, the effects of spreading losses on an echo are probably not a prime information-bearing echo features by themselves. The time-variant effects that were found in the model echoes studied here after spreading losses were removed could be more informative than the spreading losses since they were found to depend on all three foliage parameters. No information is available in the literature at the time of writing as to whether bats would be able to sense different time-variant behaviors within an echo waveform. It has been shown, however, that bats can distinguish smooth and rough echo waveforms [12]. Detecting time-variant changes in an echo waveform could possibly be handled through mechanisms that are similar to detecting the ups and downs in a rough waveform.

In the current work, echoes were simulated within a 20 kHz frequency band that is similar to the strongest harmonic of greater horseshoe bats. This is much narrower than the 115 kHz bandwidth that has been previously used for the classification of vegetation echoes [14, 15]. The results obtained here hence demonstrate that even bat species with fairly “narrow-band” biosonar signals could already have access to detailed information about complex vegetation environments without the need for the high degree of precision that large pulse bandwidths can convey [14, 29].

The simulation model studied here has been simplified by neglecting properties of natural foliages such as variable leaf geometries, acoustic shading of one leaf by another, multipath sound propagation across multiple leaves, and inhomogeneities in the spatial distribution of the leaves. It remains to be seen to which extent these factors could effect the characteristics of foliage echoes. Previous findings suggest that adding clusters to the spatial leaf distribution improves the goodness of fit between real data and simulation [14], but a full investigation of the role of leaf inhomogeneity still needs to be undertaken. Similarly, it should be investigated if alternatives to the parameter estimation approach used here suffer from the same limitation on the number of parameters that can be estimated simultaneously or if it would be possible to arrive at accurate estimates for all parameters of an unknown foliage. Sequential estimation could be a candidate methods for achieving this, since it has been demonstrated to add to performance in bioinspired classification of foliage echoes [30]. Bats could even control their motions to enhance the encoding of sensory information on the foliage [31]. If indeed all three foliage parameters considered here could be estimated without prior knowledge, it would give bats many more opportunities to master the demanding sensory task associated with navigation in complex natural environments.
